# MRI/Fluorescence bimodal amplification system for cellular GSH detection and tumor cell imaging based on manganese dioxide nanosheet

**DOI:** 10.1038/s41598-018-20110-z

**Published:** 2018-01-29

**Authors:** Dandan Yuan, Lairong Ding, Zhaomei Sun, Xuemei Li

**Affiliations:** 1 Center of Cooperative Innovation for Chemical Imaging Functional Probes in Universities of Shandong, College of Chemistry, Shandong Normal University, Jinan, 250014 P. R. China; 2 Shandong Provincial Key Laboratory of Detection Technology for Tumor Markers, School of Chemistry and Chemical Engineering, Research Institute of Biochemical Analysis, Linyi University, Linyi, 276005 P. R. China

## Abstract

Here, we report a novel magnetic resonance imaging (MRI)/fluorescence bimodal amplification platform for the detection of glutathione (GSH) on the basis of redoxable manganese dioxide (MnO_2_) nanosheets, which can be readily applied as a DNA nanocarrier, fluorescence quencher, and intracellular GSH-activated MRI contrast agent. The binding of aptamers that absorbed on the MnO_2_ nanosheets to their target can facilitating the endocytosis of target-nanoprobes. Once endocytosed, the MnO_2_ nanosheets can react with cellular GSH, resulting in the disintegration of nanosheets to generate plenty of Mn^2+^ ions for MRI and releases the primers which were adsorbed on the MnO_2_ nanosheets. Then the rolling circle amplification (RCA) reaction was initiated to amplify the fluorescence signal. In addition, after treatment with GSH, the MnO_2_ nanosheets were reduced and then most of the fluorescence was recovered. Therefore, this MnO_2_ nanoprobe exhibits excellent selectivity, suggesting a potential detection platform for analyzing the glutathione level in cells.

## Introduction

Glutathione (γ-L-glutamyl-L-cysteinyl-glycine, GSH) is the most widespread nonproteinthiol species which plays crucial role in defense against toxins and free radicals within mammalian cells^1,2^. GSH as an endogenous antioxidant is present almost exclusively in its reduced form under normal conditions. It has been proved that the level of GSH is associated with many diseases, including cancer, Alzheimer’s disease, osteoporosis, AIDS, atherosclerosis, and diseases caused by aging^3–6^. Therefore, develop a rapid, simple, sensitive methods for detection of GSH in living systems has become an important subject of current chemical research.

Up until now, there are various analytical techniques to use for GSH detection, such as fluorescence spectroscopy^7^, colorimetric assay^8^, magnetic resonance spectroscopy^9^, surface enhanced Raman scattering (SERS)^10^, enzyme-linked immunosorbent assay^11^, electrochemiluminescence (ECL)^12^, and high performance liquid chromatography (HPLC)-based separation followed by electrochemical detection^13^. However, these conventional methods that were developed early still encounter several challenges, such as tedious electrode modifications, high cost, low sensitivity. So, this issue has triggered recent development of synergistic combination of multiple experiment techniques.

Here, we report a MRI/fluorescence bimodal platform strategy for tumor cell imaging to reach the detection of GSH. Molecular magnetic resonance imaging (MRI) approaches is indeed extensively used in medical diagnostics. MRI has become a critical technology for chemists continue to devise new types of MRI contrast agents. The MRI/fluorescence dual-modal detection of GSH provides a solution able to overcome some issues of poor sensitivity and penetration. These two kinds of detection methods have their own advantages in different aspects. The fluorescent methods require the special synthesis of fluorescent materials and the fluorescence signal has poor tissue penetration. But the fluorescence signaling has the capacity for single-cell sensitivity and provide the fluorescence-guided surgical procedures by video scope^14^. On the contrary, MRI is particularly attractive and currently in development and clinical testing because of its noninvasive imaging^15^. It uses nonionizing radiation facilitating deep tissue imaging to provide high spatial resolution, while diagnosis can be difficult in areas where diseased and healthy tissues are of similar signal intensities^16^. In recent years, the application of nanosheets were more and more deeply, especially in the area of luminescent sensing^17–19^. The MnO_2_ nanosheets exhibit unique features favorable for absorb and delivery. The reduced GSH used as an antioxidant can result in the disintegration of the MnO_2_ nanosheets. In our strategy, the Mn^2+^ that generated through the reaction between the GSH and MnO_2_ nanosheets can serve as MRI contrast agent to enhance the protons’ transverse and longitudinal relaxation time. So, it provides a convenient to develop a platform with active MRI contrast agents and fluorescence signals.

In addition, signal amplification methods based on rolling circle amplification (RCA)^20,21^, have been adopted to improve fluorescence signals in our strategy. As we all know that RCA is a proven DNA replication technique that can be exploited as a powerful signal amplification tool^22,23^ because of the ability to generate RCA products with thousands of tandem DNA sequence repeats^24^. What is more, MnO_2_ nanosheets as an ultrathin two-dimension semiconductors, due to its high specific surface area and superior light absorption capability have drawn an increasing amount of attention in bioanalysis, cell imaging, and drug delivery^25–29^. And that, through the random physisorption of DNA aptamers on MnO_2_ nanosheets, the specific recognition of target cells has been achieved.

## Results and Discussion

### Principle of the sensing strategy

**Figure 1:**
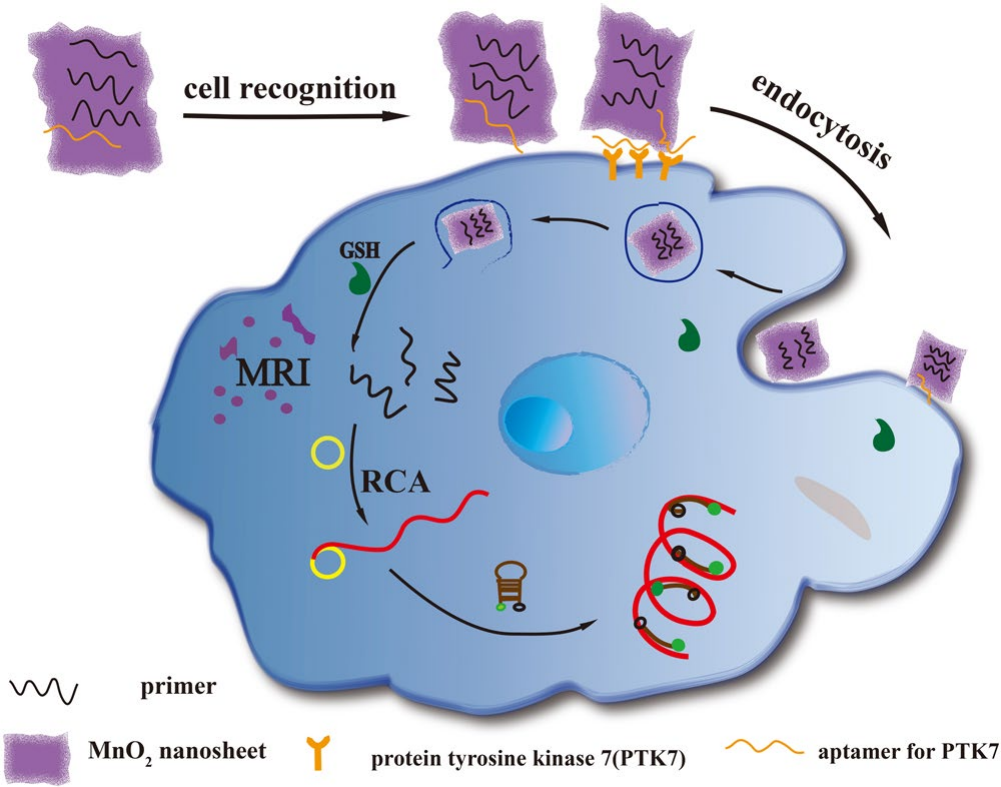
Schematic illustration of MnO_2_ nanosheets−nanoprobe for MRI/Fluorescence.

The working principle of our design is illustrated in Fig. 1. In this design, MnO_2_ nanosheets act as an oxidant to reduce the level of intracellular GSH and as a nanocarrier for primers of RCA. The fluorescence labeled ssDNA was quenched by MnO_2_ nanosheets once adsorbed on the nanosheets. Also, Mn atoms in MnO_2_ nanosheets are coordinated in octahedral geometry to six oxygen atoms and shielded from aqueous environment, making no contribution to the protons’ longitudinal or transverse relaxation^30^. Thus, compared with free Mn^2+^ ions, the MnO_2_ nanosheets is a low T1- or T2-weighted contrast agent^25^. Therefore, the redoxable MnO_2_ nanosheets as the fundamental component of the nanoprobe with multiple function, such as fluorescence quencher, carrier of target specific aptamer, and intracellular GSH activated MRI contrast agent. So we can say that, compared with other delivery carrier like Au nanoparticles or quantum dots, MnO_2_ nanosheets have multiple roles. In the presence of target cells, MnO_2_ nanosheets can react with intracellular GSH, resulting in the disintegration of the nanosheets and hence complete release of primers which were adsorbed in the MnO_2_ nanosheets. And then, under the action of Phi29 DNA polymerase, the RCA reaction was initiated by adding circular DNA template (CDT, the circular template used in this study; its sequence is given in Table S1 in supporting information) and deoxynucleotides (dNTPs). The long ssDNA were produced *in situ* through RCA process for the attachment of a large number of hairpin DNA probes. The modified hairpin DNA probes (the FAM modified on the 5′ end and the BHQ1 modified on the 3′ end, respectively) were then measured with a fluorescence spectrometer.

### Characterization of the MnO_2_ nanosheets

To construct the MnO_2_ nanosheets−nanoprobe, MnO_2_ nanosheets were prepared by ultrasonicating bulk MnO_2_, synthesized using H_2_O_2_ to oxidize MnCl_2_ in the presence of tetramethylammonium hydroxide. The results from transmission electron microscopy (TEM) indicated that as-prepared MnO_2_ presented a sheeted structure (Fig. S1). The MnO_2_ nanosheets was identified by UV/Vis absorption with a peak centered at 360 nm and the surface composition identified by energy dispersive spectrometer (EDS). The size of MnO_2_ nanosheets about 70% centered at 50–160 nm and as-prepared possessed a ζ-potential value of −32.5 mV.

### Quenching effect of the MnO2 nanosheets

Before preparing the nanoprobe of nanosheet-aptamers/primers, based on the excellent fluorescence quenching ability of MnO_2_, we first tested the quenching effect of the MnO_2_ nanosheets by fluorescence analysis^31^. As shown in Fig. S2A, the quenching efficacy was increased with the increase of MnO_2_ nanosheets volume. MnO_2_ nanosheets were easy to coagulate when the volume was too high, so we choose the 20 μL as the optimal design. GSH as a reductant could make the MnO_2_ nanosheets dissolve into Mn^2+^ ions and result in the fluorescence of FAM recovering gradually (Fig. S2B). To determine if the MnO_2_ nanosheets could protect the ssDNA from the interaction with the circular-DNA template (CDT), we examined the fluorescence of DNA1 treated with CDT and MnO_2_ nanosheets. As shown in Fig. S3, the MnO_2_ nanosheets restrained the interaction of the ssDNA and CDT. This indicated the protection of the MnO_2_ nanosheets to the ssDNA.

### Sensitive detection of GSH

**Figure 2:**
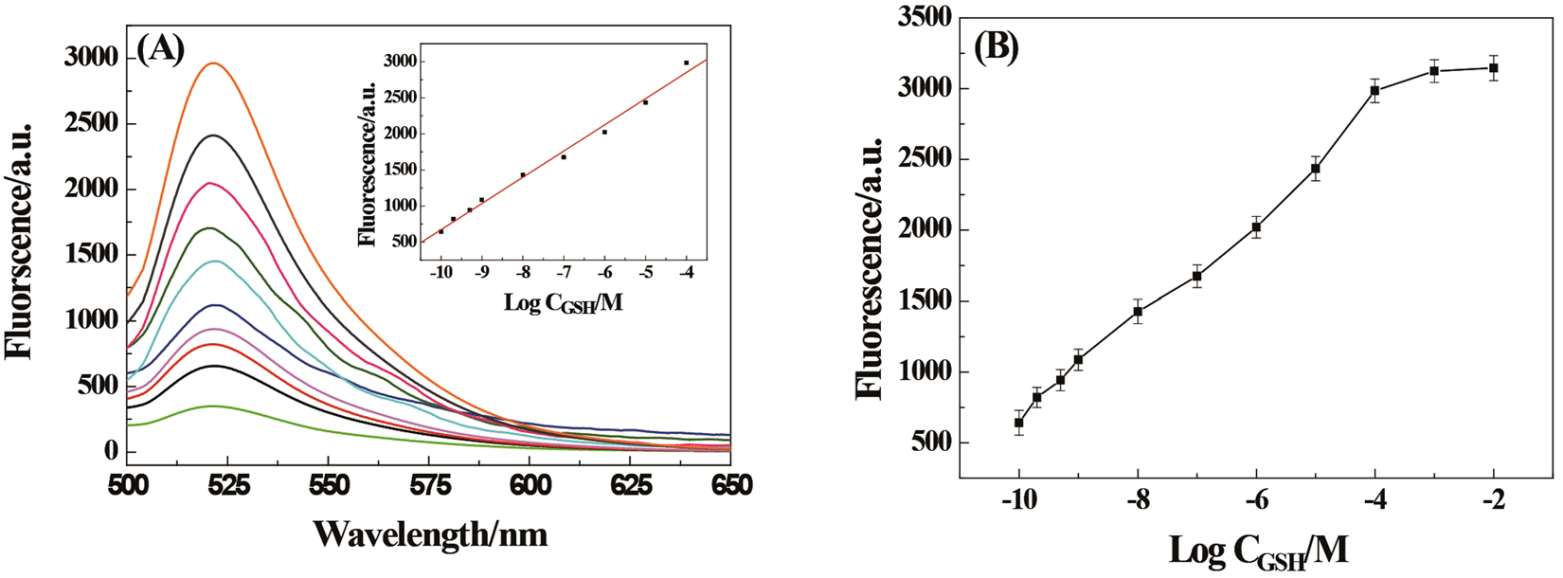
(**A**) Fluorescence spectral responses to GSH of varying concentrations *in vitro* (excitation wavelength: 488 nm). Inset: The corresponding calibration curve of the fluorescence intensity versus the concentration of GSH. (**B**) Fluorescence intensity as a function of logarithmic concentration of GSH between −10 and −2, respectively.

To demonstrate the analytical utility of the proposed approach, we employed a hairpin DNA as a molecular beacon (MB) to enable fluorescent detection of RCA products. This method takes advantage of the sensitivity of fluorescence detection, along with the unique homogeneous assaying capability offered by MB probes^32^. The sequence of the chosen hairpin DNA, was embedded into CDT and so, the resultant RCA products contain repeating sequence units that are complementary to hairpin DNA. The sensitivity of the present system was investigated under the optimum conditions upon addition of different concentrations of GSH as shown in Fig. 2A. The fluorescence intensity increased with the increased of GSH. With the measurement of the normalized fluorescence intensity in the 520 nm, the change of the intensity was quantitatively analyzed with the concentration of GSH. In the range from 1.0 × 10^−10^ − 1.0 × 10^−4^ M, the normalized fluorescence intensity was a good linear fit to the logarithm of GSH concentration, with the regression coefficient of the linear curve was R^2^ = 0.991. The assay has a limit of detection (LOD, defined as 3σ, σ = standard deviation of the blank samples) of 10 pM, which is more sensitive than previously reported detection of GSH^33^.

### Signal amplification of RCA

As shown in Fig. S4A, the control experiment without RCA reaction was carried out to prove the signal amplification of the RCA strategy. Without the GSH, the fluorescence intensity of the ssDNA were quenched mostly, so a weaken fluorescence signal was obtained. In the absence of CDT, the RCA reaction did not come up even the GSH was present. There were very small peaks in the spectra, which might be attribute to the complete release of fluorescence-labed ssDNA from the MnO_2_ nanosheets. When GSH was present, it interacted with MnO_2_ nanosheets, leading to the primer free to hybridize with the CDT. And then the RCA reaction was carried out to generate a long ssDNA that hybridized with a quantity of hairpin DNA. Each primer would have created a long ssDNA. The longer the ssDNA, the more probe tags were attached to it. In the RCA reaction, the length of the product is related to the reaction conditions, such as concentration of polymerase, dNTPs, reaction temperature and time. To control the length of RCA product consistently in all assays, the RCA reaction was ajusted for 1.5 h in the same reaction conditions. The intensity of the fluorescence signal reflects the amount of signal DNA in this detection strategy. The RCA products in solution as final results were analyzed using 1% agarose gel and stained with EB (Fig. S4B). A significant amount of RCA products was produced when primers was incubated with CDT, dNTPs and phi29 (lane 1). In addition, RCA products were not observed when CDT was omitted (lane 2). The results manifest that RCA was successfully performed.

### Selectivity and cytotoxicity of the present strategy

**Figure 3:**
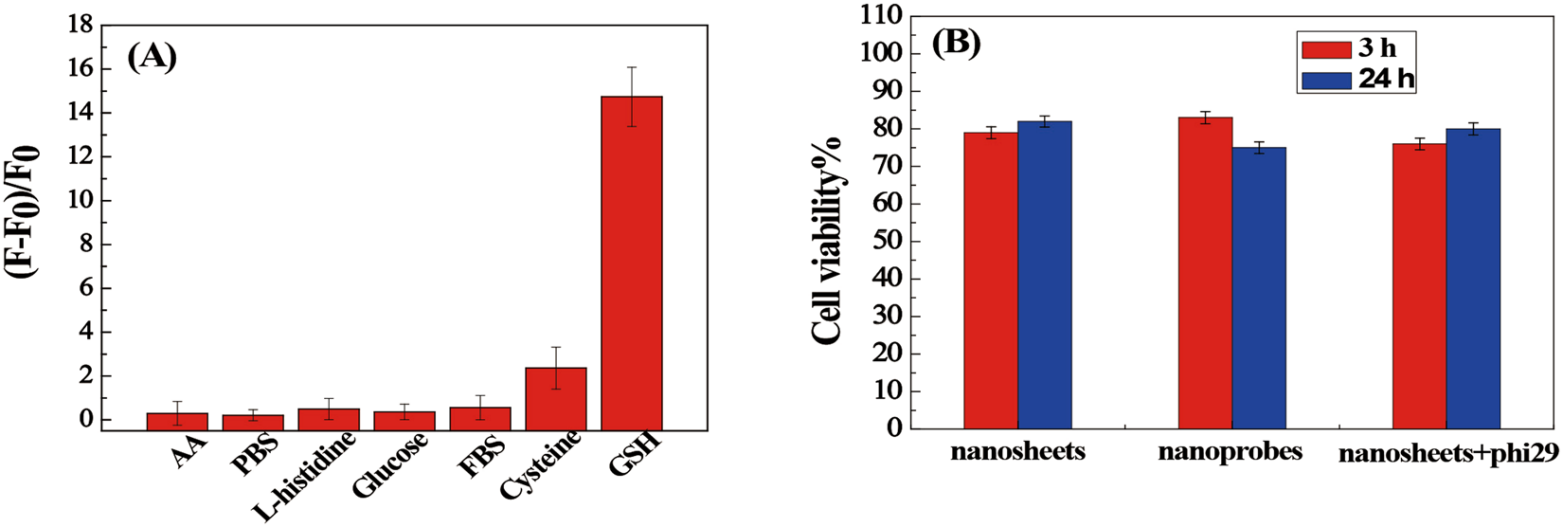
(**A**) Fluorescence response of nanoprobes in the presence of different biomolecules (concentration: 10 mM each; FBS: 10%). F0 and F represent the fluorescence intensity of nanoprobes in the absence or presence of biomolecules, respectively. (**B**) Viability of CEM cells after incubation with MnO_2_ nanosheets or MnO_2_-nanoprobe for different time periods.

Interference in GSH detection by other thiols and amino acids has been tested in detail. Based on their molecular weights and isoelectric points, some proteins were chosen as possible interference, such as fetal bovine serum (FBS), L-histinide, glucose, ascorbis acid (AA) and so on. As shown in Fig. 3A, even at a concentration as high as 10 mM, the signals from the interferences are very low and close to the background. Although cysteine (Cys) also can cause fluorescence increase, their concentrations (micromolar levels) is remarkably lower than the concentrations of GSH (millimolar levels) in biological systems^34^. This indicated that the fluorescence analytic system based biosensor has a good selectivity for the detection of GSH from other proteins.

Cytotoxicity of MnO_2_ nanosheets or MnO_2_-nanoprobe was also evaluated by CCK-8 assay, and results showed that about 80% of cells remain alive in the presence of MnO_2_ nanosheets or MnO_2_-nanoprobe (Fig. 3B). This result indicates that the nanosheets have good biocompatibility in the application and the nanocomposite has low cytotoxicity.

### Flow cytometric and cell imaging with MnO_2_-nanoprobe

**Figure 4:**
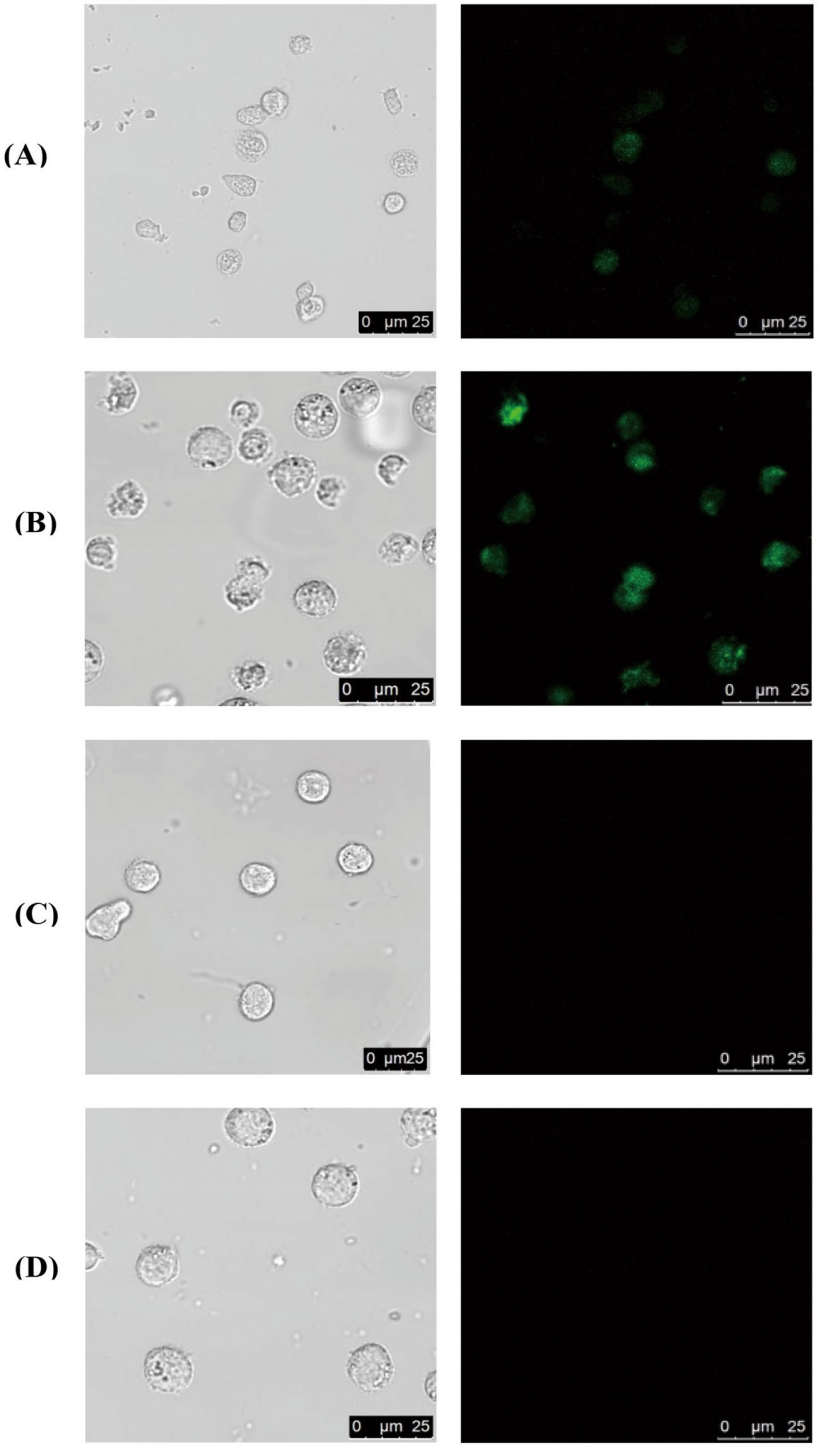
Confocal imaging of cells treated with MnO_2_nanosheet. (**A**) CEM+, RCA−, Sgc 8+; (**B**) CEM+, RCA+, Sgc 8+; (**C**) Ramos+, RCA+, Sgc 8+; (**D**) CEM+, RCA+, Sgc 8−.

After verification *in vitro*, we explored the imaging potential of hairpin DNA after RCA in single cells. To construct the specific nanoprobe, Sgc8 (60 bases) targeting protein tyrosinekinase 7 (PTK7)^35^ that with an extra 19 bases at the 5′-terminus was chosen as an aptamer in this strategy. We examined the fluorescence of PTK7-positive CCRF-CEM cells and PTK7-negative Ramos cells treated with the nanoprobe to determine the target recognition. To identify the effect of RCA, experiment was carried out for the system with and without RCA by confocal laser scanning microscope (CLSM). As shown in Fig. 4A and B, the fluorescence signal obtained after RCA was stronger than that without RCA. The reason for this result may be that the long ssDNA as the RCA products attached a mass of fluorescence-labeled hairpin DNA. When the RCA was absent, the hairpin will not interaction with the long ssDNA, so we obtained a weak fluorescence signal from the fluorescence-labeled DNA1 which were adsorbed on the MnO_2_-nanosheets. CCRF-CEM cells exhibited a brighter green fluorescence signal than Ramos cells under the same conditions (Fig. 4C). The CEM cell do not show obvious fluorescence when we use other aptamer replacement sgc8 aptamer (Fig. 4D). These results proved that the fluorescence response of the MnO_2_-nanoprobe was target cell-specific and aptamer-specific activatable.

The interaction of the MnO_2_ -nanoprobes and target cells were further tested by flow cytometry. As shown in Fig. S5A, the CEM cells treated with RCA presented a higher fluorescence signal compared to those treated with nanocomplexes containing of ATP aptamer or without RCA. However, the MnO_2_-nanoprobes could not act on the PTK7-negative cells, such as Ramos cell, and in spite of with or without RCA (Fig. S5B). These results further demonstrated that the fluorescence activation of the MnO_2_-nanoprobe was specific to target cells. The replacement of the aptamer and target cells could not enhance the fluorescence signal. The amplification of RCA in this system was very obvious.

To account for the results above, the interaction between MnO_2_-nanoprobes and target cells may be attributed to the following facts: (1) sgc8 aptamers that adsorbed on MnO_2_ nanaosheets could facilitate the endocytosis of MnO_2_-nanoprobes after binding to its protein on the surface of the target cell. (2) Endocytosed nanocarriers are reduced to Mn^2+^ ions by intracellular GSH, and released the primers leading to the RCA^30^. And then the RCA products as a fluorescence activation inspire the fluorescence of the hairpin DNA.

### MRI detection

**Figure 5:**
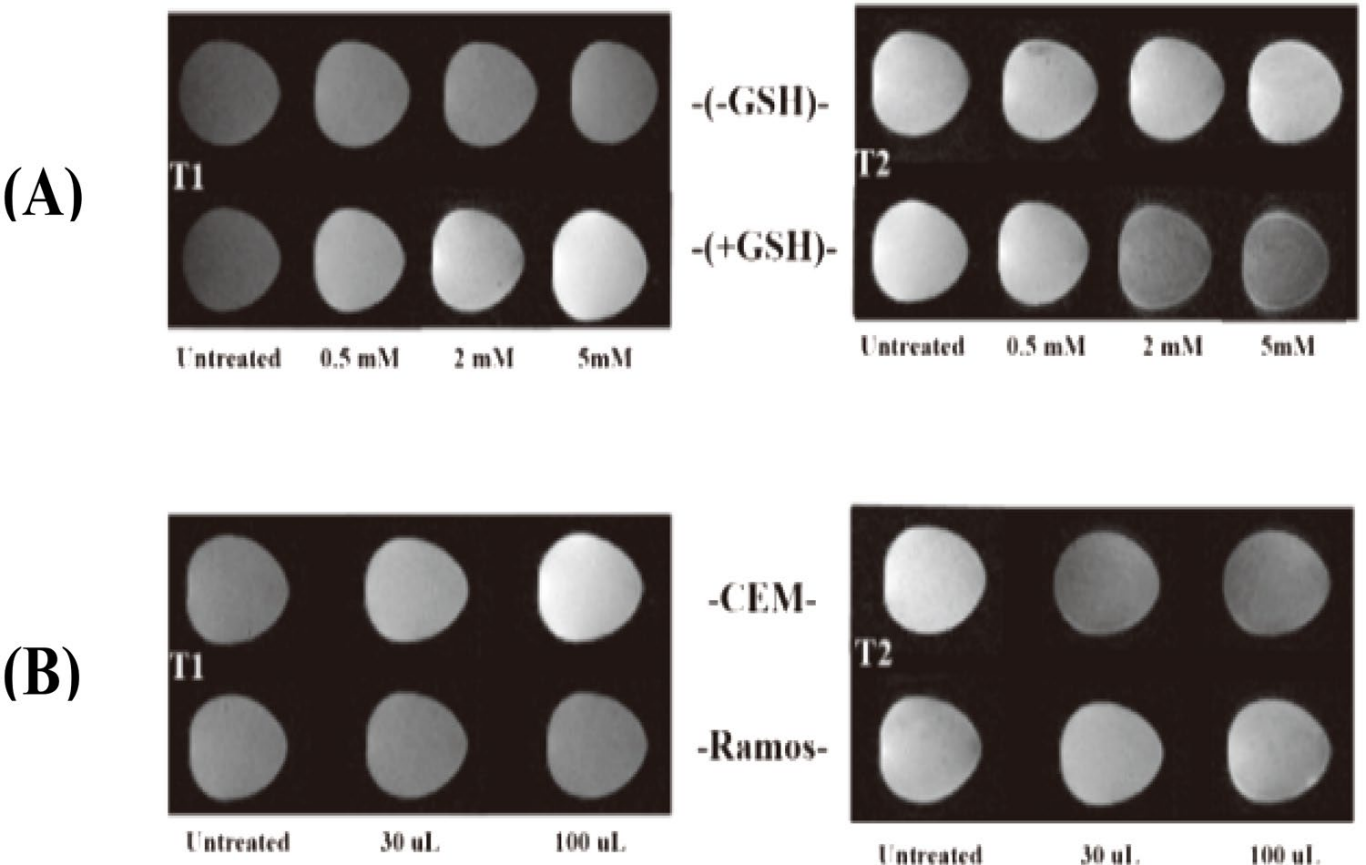
(**A**) T1-weighted and T2-weighted MRI images of MnO_2_ nanoprobes solution treated with GSH. (**B**) T1-weighted and T2-weighted MRI images of CCRF-CEM cells and Ramos cells incubated with MnO_2_ nanoprobes at various volume.

To determine the mechanism of the MnO_2_ nanosheet as a MRI contrast agent, T1-weight and T2-weight MRI results of MnO_2_ before and after interaction by GSH was examined. When MnO_2_ nanosheets were reduced to Mn^2+^ by GSH, a large amount of Mn^2+^ ions were act on the MRI to enhance the longitudinal relaxation times and protons’ transverse. The contents of Mn in each CEM cells treated with nanoprobe were detected by inductively coupled plasma mass spectroscopy (ICP-MS) and were 0.03825 pg. And then a dramatic enhancements in T1-weight and T2-weight MRI contrast signals was obtained with GSH at different concentrations (Fig. 5A). We also evaluated the effectiveness of the MnO_2_-nanosheets for cellular MRI by examination of Ramos cell and CEM cell with different volume of nanoprobes. Through the comparison of CCRF-CEM cell and Ramos cell after the treatment of MnO_2_-nanoprobes, the results indicate that the CEM cell shows higher T1-weight and T2-weight MRI contrast signals than Ramos cell under the same conditions (Fig. 5B). This result may be attributed to the target-cell recognition of nanoprobes and the reduction of MnO_2_ by GSH.

## Conclusions

In conclusion, we have contruct a sensitive MnO_2_ nanoprobe for target cell amplifying fluorescence signal and intracellular GSH-activated MRI. The MnO_2_ nanosheets as the basic component of nanoprobes, the main effect of it in our design is that MnO_2_ nanosheets act as (1) a nanocarrier to adsorb aptamers or primers, (2) fluorescence quencher to quench fluorescence labeled on the DNA, (3) intracellular GSH-activated MRI contrast agent. To indicate the cell-specific of the MnO_2_ nanoprobes, we have demonstrated the fluorescence signal of CCRF-CEM cell and Ramos cell that was treated with the nanoprobes. We have achieving amplify detection of GSH with a detection limit of 10 pM and excellent selectivity thought RCA. We have also demonstrated the mechanism of intracellular GSH activated MRI contrast agent. We believe that this MRI/Fluorescence dual-modal amplification detection system that with a high sensitivity and selectivity will have wide potential applications in cancer cell image.

## Materials and Methods

### Apparatus

Zeta potential and dynamic light scattering were measured on the Malvern ZetasizerNano ZS90 (Malvern Instruments, Ltd., Worcestershire, UK). Confocal imaging was performed at Leica TCS SP5 II laser scanning microscopy with an HCX PL APO 20×/0.85 Corr CS objective, using 488 nm as the excitation wavelength. All fluorescence measurements were carried out on a F4600 fluorometer (Hitachi, Japan). UV/Vis absorption spectra were obtained by a Cary 50 Series Spectrophotometer (Varian, Australia). The structure of the MnO_2_ and their surface composition were acquired using a transmission electron microscopy (TEM) instrument (JEM-2100, Hitachi, Japan). MRIs were measured by T7 MRI instrument (Bruker Biospec). The flow cytometric analysis was analyzed on the CutoFLEX flow cytometer (Back Coulter, USA).

### Chemicals

All oligonucleotides used were synthesized from Takara Biotechnology Co., Ltd. (Dalian, China), the sequences are summarized in Supporting Information Table S1. CCRF-CEM and Ramos cells were purchased from the Boster Biological Technology Co., LTd. (Wuhan, China). Dul-becco’s modified eagle medium (DMEM) was obtained from HyClone. The Cell Counting Kit-8 (CCK-8) kit was purchased from Kang Long Biological Technology Co., Ltd. (Shanghai, China). Tetramethylammoniam hydroxide, DL-1, and L-glutathione (GSH) were purchased from Sigma-Aldrich and used as received without further purification. Manganese chloride tetrahydrate (MnCl_2_•4H_2_O) were obtained from Aladdin Industrial Inc. (Shanghai, China). deoxyribonucleoside 5′-triphosphates (dNTPs), Phi29 DNA polymerase and 10 × phi29DNA polymerase reaction buffer and were purchased from Thermo Scientific. Hydrogen peroxide (H_2_O_2_, 30 wt%) and other regents were all of analytical grade, purchased from Sinopharm Chemical Reagent Co., LTd. (Shanghai, China) and used as received unless otherwise mentioned.

All solutions were prepared using ultrapure water, which was prepared through a Millipore Milli-Q water purification system (Billerica, MA, USA), with an electrical resistance >18.3 MΩ.

### Preparation of MnO_2_ nanosheets

Manganese dioxide nanosheets were synthesized according to previous reports. Typically, 20 mL of a mixed aqueous solution of 3 wt% H_2_O_2_ and 0.6 M tetramethylammonium hydroxide were prepared. Then the solution was added to 10 mL of 0.3 M MnCl_2_ solution quickly within 15 s. The solution became dark brown immediately indicating that Mn^2+^ was oxidized to Mn^4+^ and then the resulting dark brown suspension was stirred vigorously overnight in the open air at room temperature. The as prepared bulk manganese dioxide was centrifuged at 2000 rpm for 20 minutes and then washed with water and methanol. After that, the bulk manganese dioxide was dried in a drying oven at 60 °C and kept in a centrifuge tube for further experiments. To prepare the MnO_2_ nanosheets, 10 mg bulk manganese dioxide was dispersed in 20 mL water and ultrasonicated for at least 10 h. Then, the dispersion was centrifuged at 2000 rpm for 30 min, and the supernatant was kept for further use.

### Preparation of MnO_2_ nanosheet-ssDNA nanocomplex or nanoprobe

The physisoprtion of ssDNA on MnO_2_-nanosheets was carried out by mixing 15 μL of MnO_2_ nanosheets and 10 μL of ssDNA or aptamer (1 μM) for 30 min followed by the addition of 75 μL of HEPES buffer (20 mM, pH 7.2, containing 150 mM NaCl and 2 mM MgCl_2_) for 30 min. Then, the fluorescence measurement of MnO_2_ nanosheet-ssDNA and equivalent ssDNA was performed on a F4600 fluorometer (Hitachi, Japan).

### Cell Culture and buffer

CCRF-CEM and Ramos cells were purchased from Boster Biological Technology Co., LTd. (Wuhan, China) and were cultured in RPMI 1640 media (Gibco) supplemented with 10% (v/v) fetal bovine serum (Gibco), 100 U/mL penicillin/streptomycin at 37 °C in 5% CO_2_ atmosphere. Dulbecco’s phosphate- buffered saline (DPBS) without Ca^2+^ and Mg^2+^ was used to wash cells. Approximately one million cells dispersed in medium were centrifuged at 1000 rpm for 3 min, washed and then redispersed in medium (1 mL).

### Gel electrophoresis

Agarose gels were prepared by mixing agarose (2%) with TAE buffer and microwaving the resultant mixture for 1 min, and then add the ethidium bromide gel stain before the gel solidified. Transferred the solidified gel to an electrophoresis apparatus filled with TAE buffer. The loading buffer was added to each sample before being loaded into a well, followed by the conduction of gel electrophoresis at 120 V for 50 min. Finally, the results of gel images were visualized using a UV transilluminator.

### GSH recognition and RCA

Typically, the MnO_2_ nanosheet-ssDNA nanocomplex or nanoprobe were prepared as mentioned for further use. Then, 10 μL of the above stock solution was transferred into a 200 μL micro centrifuge tube. Subsequently, 1 μL of GSH stock solution (0–100 μM) and 1 μL of CDT (1 μM) were added. And then, 0.5 μL phi29 DNA polymerase, 5 μL 10× phi29 DNA polymerase buffer (330 mM Tris-acetate, pH 7.9 at 37 °C, 100 mM Mg-acetate, 660 mM K-acetate_,_ 1% (v/v) Tween 20, 10 mM DTT), 2.5 μL dNTPs (with a final concentration of 500 μM) were introduced to the above mixture (total volume: 50 μL). The reaction mixture was incubated at 37 °C for 1.5 h before heating at 65 °C for 15 min. Finally, 5 μL of RCA products from the above reaction mixture was added into 85 μL PBS (20 mM, pH 7.5, 150 mM NaCl, 5 mM MgCl_2_) containing 1 μM hairpin DNA as a signal DNA in a cuvette with a constant temperature at 30 °C for 1 h.

The procedure for other biological molecules detection was similar to that for the GSH detection.

### Fluorescence spectrum measurements

All fluorescence measurements were carried out on a F4600 fluorometer (Hitachi, Japan) with a 200 μL quartz cuvette. Under the condition of slit width was 5 nm, the emission spectra were obtained by exciting the samples at 488 nm and scanning the emission from 500 nm to 650 nm.

### Flow cytometric and confocal imaging analysis

For RCA, the MnO_2_ nanosheet-ssDNA nanocomplex or nanoprobe were performed a volume of 100 μL containing 5 μL primer, 0.5 μL aptamer, 7.5 μL MnO_2_ nanosheet and 87 μL HEPES. And then, the RCA was performed in a volume of 150 μL containing 100 μL nanoprobe, 5 μL CDT, 2 μL phi29 DNA polymerase, 15 μL 10× phi29 DNA polymerase buffer (330 mM Tris-acetate, pH 7.9 at 37 °C, 100 mM Mg-acetate, 660 mM K-acetate_,_ 1% (v/v) Tween 20, 10 mM DTT), 5 μL dNTPs and 23 μL DEPC-treated water. For hairpin probe, the physisoprtion of signal DNA on MnO_2_ nanosheet was carried out by mixing 80 μL signal DNA and 40 μLMnO_2_ nanosheet for 10 min.

One million cells (CEM or Ramos) were incubated with the prepared a mixture of two different solutions as above mentioned for 4 h at 37 °C. The incubation was followed by two washes in DPBS for confocal imaging analysis.

All the wide-field or confocal laser scanning microscopy (CLSM) images were collected on the Leica TCS SP5 II microscopy with an HCX PL APO 20×/0.85 Corr CS objective. CLSM images were obtained using an exciting laser at the wavelength 488 nm and the fluorescence detection band was set to 500–650 nm for rhodamine green using photomultiplier tubes (PMT), while the bright-field images were recorded simultaneously with the CLSM images using transmission PMT. In some occasions, CLSM images and transmitted-light images were superimposed for comparison. After confocal imaging analysis, the samples were analyzed using a CutoFLEX flow cytometer.

### Measurement of intracellular manganese content

CEM cells (1 × 10^7^) were incubated with MnO_2_ nanoprobes at the volume of 1.5 mL for 3 h at 37 °C. After removing the supernatant, cells were washed twice with DPBS. Then the cell sampleS were treated with ultrasonic cell crusher. After cell lysate was removed using a 0.22-μm filter, the volume of these samples was adjusted to 3 mL. The measurement of manganese content was carried out on an inductively coupled plasma mass spectrometry (ICP-MS). The cellular manganese concentration was calculated by dividing the total manganese content by the number of cells.

### *In vitro* MRI of cells

Firstly, the T1- and T2-weighted MRI signals of MnO_2_ nanosheets at different volumes treated with or without 10 mM GSH were measured on a Bruker Biospec analyzer to verify the response of MnO_2_ nanosheets as MRI contrast agents to GSH. For the MRI of cells, CEM or Ramos cells (1 × 10^5^) were incubated with MnO_2_ nanoprobes at 0, 30 μL and 100 μL at 37 °C for 4 h. At the predetermined time, cells were washed twice with DPBS after the supernatant was removed. Then, cells were mixed with 0.8% agarose. And then, the mixture was transferred into 96-well plates for MRI.

### Cell Viability

The cytotoxicities of MnO_2_ nanosheet, MnO_2_ nanocomplex or nanoprobe for target CEM cells were evaluated by CCK-8 assay. CEM cells were seeded in a 96-well plate at a density of 5 × 10^5^ cells per well. After overnight incubation, the cells were treated with 200 μL of cell medium containing MnO_2_ nanosheets or nanoprobes incubated for 3 h or 24 h. At the preassigned time, the cell medium was then removed, and 200 μL of fresh cell medium was added, followed by the addition of 20 μL of CCK-8 solution to each well. After 4 h incubation, the optical density (OD) of each well at 450 nm was recorded on a Microplate Reader. The cell viability (%) is expressed as the percentage of (OD_test_ −OD_blank_)/(OD_control_ −OD_blank_), where OD_test_ is the optical density of the cells exposed to MnO_2_ nanocomplex or nanoprobe, OD_control_ is the optical density of the control sample and OD_blank_ is the optical density of the wells without cells.

## Electronic supplementary material

supplementary information
