# Supplementary material for: MRI/Fluorescence bimodal amplification system for cellular GSH detection and tumor cell imaging based on manganese dioxide nanosheet
Source: Sci Rep. 2018 Jan 29;8:1747. doi: 10.1038/s41598-018-20110-z (PMC5788857; doi:10.1038/s41598-018-20110-z)
Supplement: Supplementary file 1 — supplementary information [file 41598_2018_20110_MOESM1_ESM.pdf]

Supporting Information

MRI/Fluorescence bimodal amplification system for cellular GSH detection and tumor cell imaging based on manganese dioxide nanosheet

Dandan Yuan[1,2,] Lairong Ding[1,2], Zhaomei Sun[2], Xuemei Li[2]

[1]Center of Cooperative Innovation for Chemical Imaging Functional Probes in Universities of Shandong, College of Chemistry, Shandong Normal University, Jinan 250014, P.R. China.

[2]Shandong Provincial Key Laboratory of Detection Technology for Tumor Markers, School of Chemistry and Chemical Engineering, Research Institute of Biochemical Analysis, Linyi University, Linyi 276005, P. R. China.

Dandan Yuan (E-mail: yddwhxf@163.com)

Lairong Ding (E-mail: 1582785909@qq.com)

Zhaomei Sun (E-mail: anqi7903@163.com)

Xuemei Li* (xuemei_li@yeah.net)

*Corresponding author. Tel: 86-539-8766867; Fax: 86-539-8766867

E-mail address: xuemei_li@yeah.net (Xuemei Li)

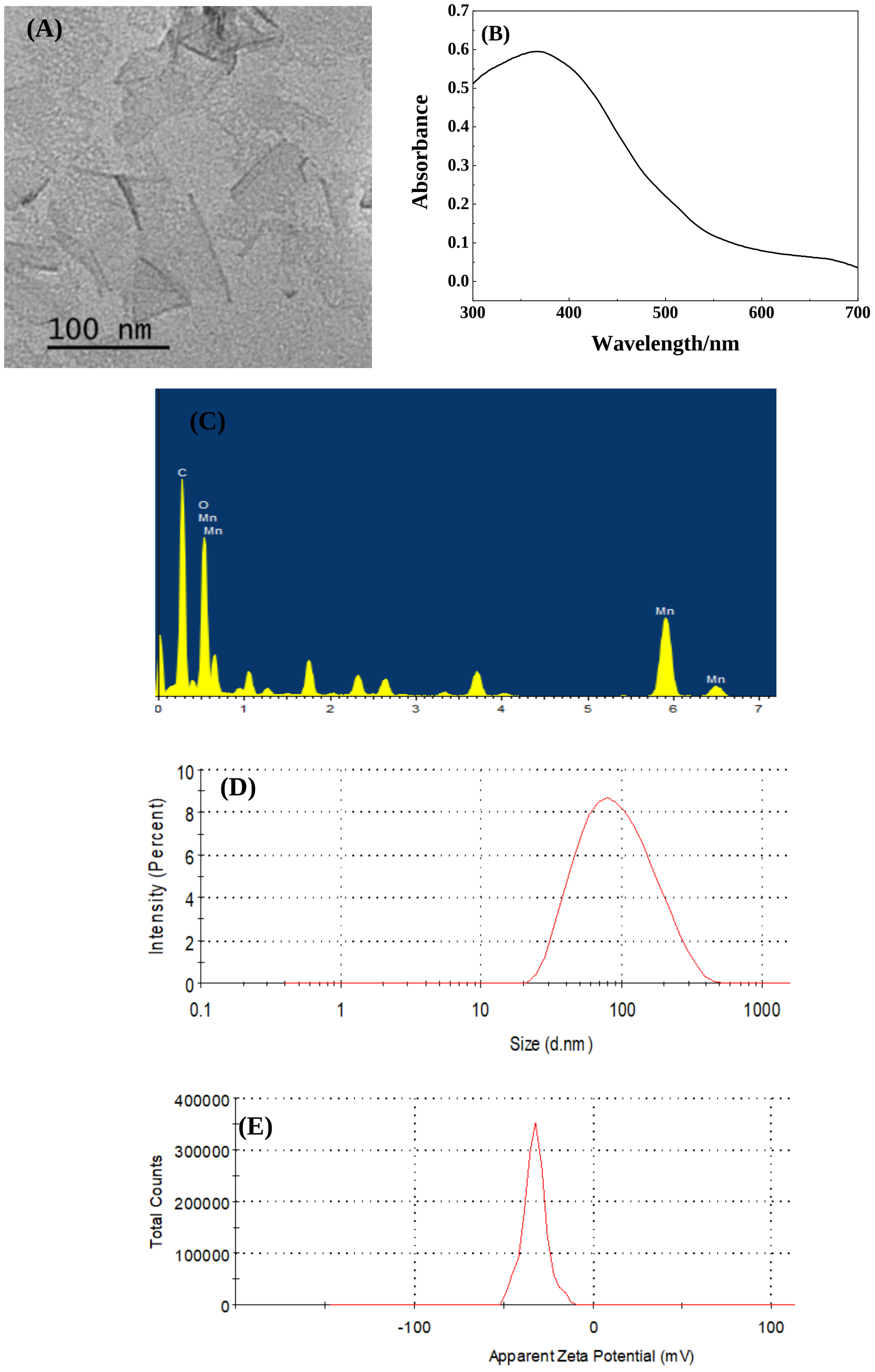

Fig S1 (A) TEM, (B) UV−vis absorption spectrum, (C) EDS, (D) size analysis and (E) zeta potential analysis characterization of MnO_2 nanosheets.

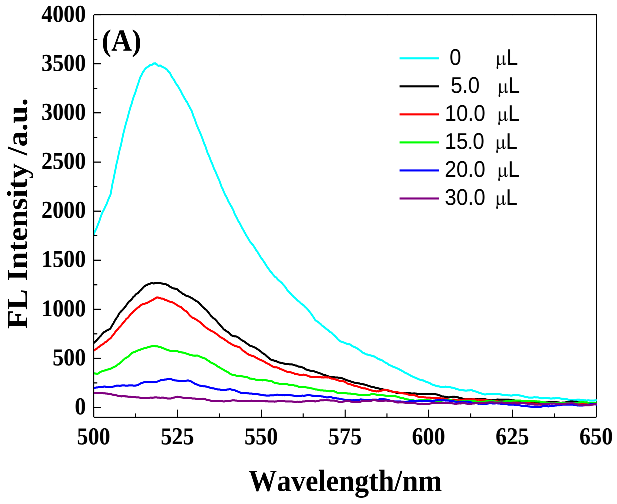

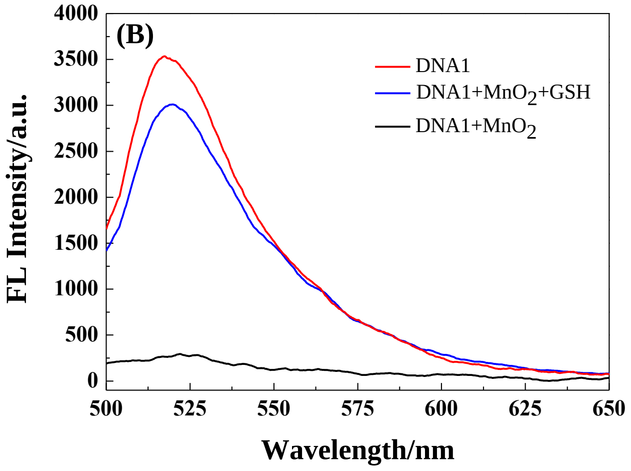

Fig S2 (A) Quenching effect of the MnO_2 nanosheets on 100 nM ssDNA. (B) Fluorescence intensities of the ssDNA (100 nM) with MnO_2 nanosheets and GSH (excitation at 488nm).

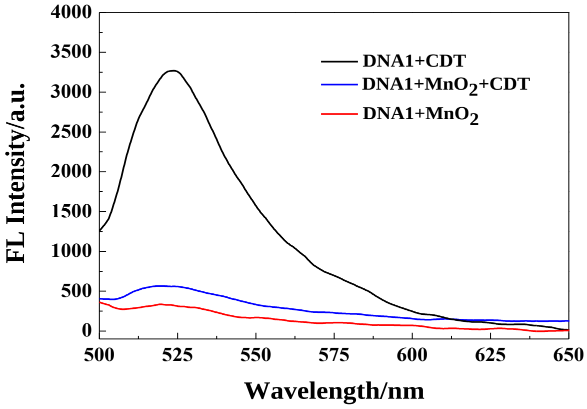

Fig S3 The protection of the MnO_2 nanosheets to the ssDNA (excitation at 488nm).

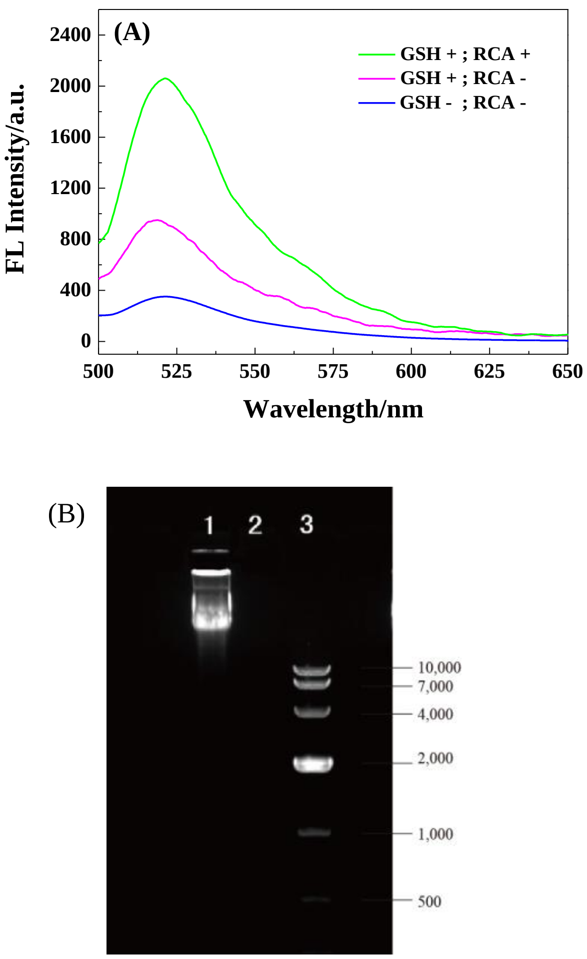

Fig S4 (A) FL spectra of DNA1 obtained from RCA detection of GSH. (B) The image of gel electrophoresis experiment. Lane (1): the RCA product, lane (2): RCA reaction without CDT, and lane (3): DL10000bp DNA markers.

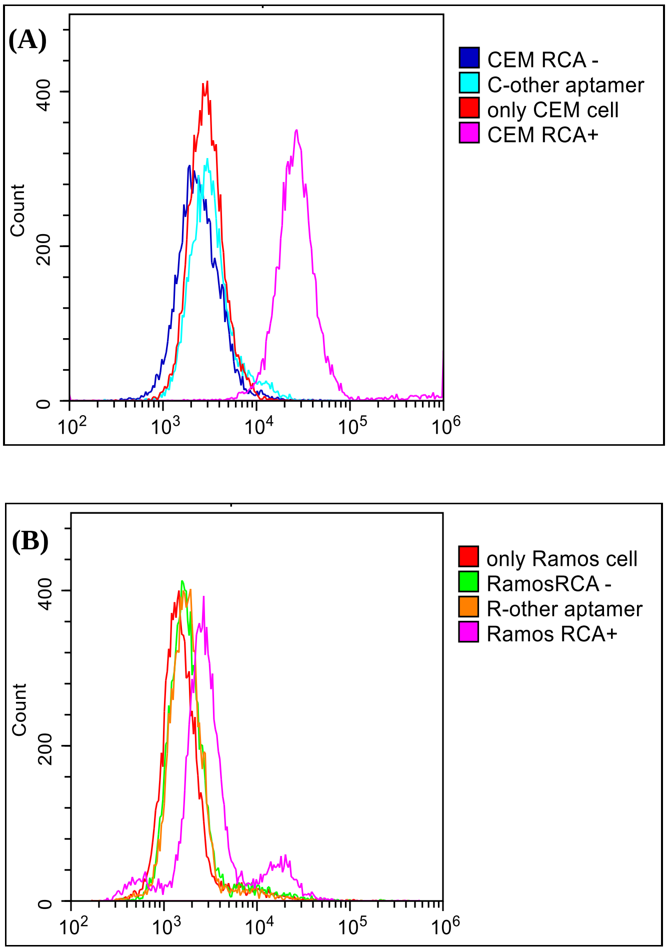

Fig S5 Flowcytometric results of the MnO_2-nanoprobe in CEM (A) and Ramos cells (B).

Table S1 DNA sequences used in the present experimental.

Name	Sequences
Aptamer-Sgc8	5’-ATTAT TATTA TTATT ATTAT TCTAA CTGCT GCGCC GCCGG GAAAA TACTG TACGG TTAGA-3’
Primer	5’-CAGTC AGT CA GTAAA GGAAG-3’
Signal DNA	5’-FAM-CGCTCCTTTC GTTTC CTTGA AACTTGAGCG-Dabcyl-3’
DNA 1	5’-FAM-CAGTC AGTCA GTAAA GGAAG-3’
Circular-DNAtemplate (CDT)	5’-ACTGA CTGCC TTCTT CTTTC GTTTC CTTGA AACTT TTTCC CTTCC TTTAC TGACT GACTG-3’
Aptamer	5’-FAM-ACCTGGGGGAGTATTGCGGAGGAAGGT-3’
